# Global clinical trials on stem cell therapy for autoimmune diseases: trends and future directions

**DOI:** 10.3389/fimmu.2025.1616231

**Published:** 2025-07-24

**Authors:** Yanhao Chen, Xiang Li, Jiao Zhang, Jiaqi Peng, Fugang Huang, Jie Bao, Yongsheng Fan, Shuo Huang

**Affiliations:** ^1^ The Second School of Clinical Medicine, Zhejiang Chinese Medical University, Hangzhou, China; ^2^ School of Basic Medical Sciences, Zhejiang University of Chinese Medicine, Hangzhou, China; ^3^ The Second Affiliated Hospital of Zhejiang Chinese Medical University (Xinhua Hospital of Zhejiang Province), Hangzhou, China

**Keywords:** autoimmune diseases, clinical trials, Crohn’s disease, mesenchymal stem cells, stem cell therapy

## Abstract

**Background:**

Autoimmune diseases, such as Crohn’s disease (CD) and systemic lupus erythematosus (SLE), lead to progressive multi-organ damage due to immune dysregulation and chronic inflammation. Current therapies lack efficacy and safety, often failing to sustain remission. Stem cell therapy has emerged as a promising approach for immune modulation and tissue repair. This study analyzes clinical trial trends and challenges of stem cell therapy in autoimmune diseases.

**Methods:**

Clinical trial data (2006–2025) were extracted from Trialtrove. Strict inclusion criteria were applied, restricting the analysis to interventional trials while excluding observational studies, non-autoimmune disease trials, and records with incomplete information. Descriptive statistics were used to analyze trial phases, disease types, geographic distribution, funding sources, therapeutic mechanisms, and stem cell sources, followed by a comparative evaluation of therapeutic efficacy, combination strategies, and safety profiles across autoimmune indications.

**Results:**

Of the 1,511 global trials, 244 were included after screening and cross-referencing. Most trials (83.6%) were in Phase I-II. CD (n=85), SLE (n=36), and scleroderma (n=32) were the most studied. The U.S. and China led in trial numbers. Academic institutions funded 49.2% of trials. Key therapeutic strategies included immune modulation, tissue repair via growth factors, and anti-infection/anti-proliferative effects. Disease-specific variations were noted in cell sources and administration routes.

**Conclusion:**

Stem cell therapy holds substantial promise for autoimmune disease treatment. Future efforts should prioritize technological innovation, international collaboration, and precision medicine to address current challenges and advance clinical translation.

## 1 Background

Autoimmune diseases, as a group of diverse and complex systemic disorders, are characterized by dysregulated immune homeostasis and chronic, persistent inflammation (1). This category of diseases, encompassing rheumatoid arthritis (RA), scleroderma, and inflammatory bowel disease, exhibits considerable heterogeneity, evident in the dysregulated activation of distinct immune cell subsets as well as the diverse patterns of target organ involvement. For instance, RA is marked by aberrant activation of autoreactive T and B lymphocytes, leading to persistent synovial inflammation and joint destruction, whereas scleroderma involves vascular dysfunction and fibroblast-driven fibrosis mediated by Th2-skewed immune responses (2, 3). Inflammatory bowel diseases such as Crohn’s disease (CD) and ulcerative colitis are associated with impaired intestinal barrier function and dysregulated Th cell responses (4). Systemic lupus erythematosus (SLE) is characterized by the production of autoantibodies against nuclear antigens and immune complex deposition across multiple organs (5). Psoriasis, a chronic inflammatory skin disease, is driven by dysregulated dendritic cell–T cell–keratinocyte crosstalk, particularly through the IL-23/IL-17 axis (6). Epidemiological studies indicate that autoimmune diseases currently affect more than one billion people worldwide, with an increasing incidence in developing countries due to environmental shifts, lifestyle changes, and advancements in diagnostic techniques (7, 8).

In the clinical management of autoimmune diseases, a stepwise or individualized treatment strategy is typically employed to balance acute symptom control and long-term disease management. Glucocorticoids and nonsteroidal anti-inflammatory drugs (NSAIDs) are widely used for their rapid suppression of acute inflammation, while traditional immunosuppressants and targeted biologics serve as the cornerstone therapies for maintaining disease remission and controlling disease progression (9, 10). Recently, novel small-molecule drugs, including JAK-STAT pathway inhibitors, have been integrated into treatment regimens, especially for patients with refractory disease who exhibit inadequate responses or intolerance to conventional or biologic therapies (11). Despite these advancements, treatment challenges persist. With disease progression or in complex cases, the limitations of conventional treatments become more evident. Beyond primary or secondary drug resistance in some patients, prolonged medication use leads to cumulative toxicity, including glucocorticoid-induced metabolic syndrome, biologic therapy-associated opportunistic infections, and the cardiovascular risks linked to JAK inhibitors (12–14). Moreover, current therapies can only suppress abnormal immune responses but fail to reverse established tissue fibrosis or promote functional repair, leaving patients at risk of irreversible organ dysfunction even after disease remission.

The unmet clinical demands have catalyzed the strategic deployment of stem cell therapy as an alternative approach to overcoming the limitations of conventional treatments. Mesenchymal stem cells (MSCs) are one of the most extensively studied stem cell types in clinical research due to their potent immunomodulatory and regenerative properties (15). They can regulate immune tolerance and maintain immune homeostasis by secreting soluble factors such as TGF-β, PGE2, and IDO, as well as exosomes enriched with regulatory miRNAs like miR-21 and miR-146a, which suppress excessive activation of Th1 and Th17 cells while promoting the expansion of regulatory T cells (Treg) (16–18). Additionally, MSCs possess the ability to migrate to inflamed sites in a CXCR4/SDF-1 axis-dependent manner and differentiate into functional stromal cells within the local microenvironment, thereby directly repairing damaged tissues (19). Hematopoietic stem cell (HSC) transplantation primarily achieves therapeutic effects by using high-dose immunosuppression or chemotherapy to eliminate the aberrant immune system, followed by the re-establishment of immune tolerance, which has demonstrated long-term remission potential in autoimmune diseases such as scleroderma and multiple sclerosis (20, 21). Induced pluripotent stem cells (iPSCs) can be genetically engineered to generate specific immunoregulatory cells, such as Treg or tolerogenic dendritic cells, or differentiate into functional target tissue cells, thereby enabling precise therapeutic interventions (22–24).

Despite the promising prospects of stem cell therapy in autoimmune diseases, its clinical translation still faces multiple challenges. Among these, the most significant barrier is the high cost and complexity of personalized therapy. Stem cell treatment necessitates individualized customization, including differentiation of autologous iPSCs or selection of allogeneic MSCs, as well as intricate processes like cell culture, genetic modification, and quality assurance. These processes collectively incur substantial expenses that often far exceed the cost range of traditional biologic therapies, posing a major hurdle to widespread clinical adoption (25, 26). Another key concern is the lack of robust long-term safety data. While short-term studies have demonstrated favorable tolerability of stem cell infusions, allogeneic MSCs may elicit mild immune rejection, requiring extensive long-term follow-up studies to establish clear safety benchmarks and regulatory guidelines (27). Consequently, a thorough examination of the current applications, research trajectories, and future potential of stem cell therapy across different autoimmune diseases is of great clinical importance.

To achieve this, we leveraged the Trialtrove database, which consolidates global clinical trial data, providing a comprehensive platform for tracking and evaluating the latest developments in stem cell therapy for autoimmune diseases (28–30). By analyzing the annual trends in trial registrations, geographic distribution, funding sources, cell types, administration routes, and other related factors, we can comprehensively understand research trends and implementation patterns of stem cell therapy in autoimmune diseases. Such an in-depth evaluation contributes not only to a more thorough understanding of the therapeutic potential of stem cell therapy but also offers evidence-driven insights for future clinical applications and outcome assessments.

## 2 Materials and methods

### 2.1 Data sources and selection standards

This study systematically collected global clinical trial data from the Trialtrove database, a repository renowned for its rigorously curated, comprehensive, and methodologically robust compilation of trials spanning diverse therapeutic areas and geographic regions (28–30). We selected all clinical trials registered up to January 2, 2025, that involved stem cell therapy in autoimmune diseases. A structured search strategy was implemented using the keywords “Drug Type: ‘Stem Cell Therapy’” and “Therapeutic Area: ‘Autoimmune/Inflammation’” to ensure comprehensive data retrieval. To address potential limitations associated with relying on a single database, we additionally retrieved and cross-checked relevant trial records from ClinicalTrials.gov and the EU Clinical Trials Register.

### 2.2 Inclusion and exclusion criteria

The screening process in this study strictly followed a set of predefined inclusion and exclusion criteria. The inclusion criteria were restricted to trials specifically focusing on stem cell therapy for the following autoimmune diseases: alopecia areata, ANCA-associated vasculitis (antineutrophil cytoplasmic antibody-associated vasculitis), autoimmune hepatitis, CD, dermatomyositis/polymyositis, SLE, other inflammatory arthritis, pemphigoid, primary biliary cholangitis, psoriasis, RA, scleroderma, Sjögren’s syndrome, ulcerative colitis, and vitiligo. Studies were excluded if they met any of the following conditions: (1) observational in nature rather than interventional clinical trials; (2) involving incompatible diseases rather than autoimmune conditions; (3) conducted outside the defined timeframe of 2006 to 2025; or (4) classified as trials with unknown phases (31).

### 2.3 Data extraction and statistical analysis

Data extraction was conducted by two independent investigators following a predefined protocol to ensure both consistency and accuracy. Key characteristics of all included trials were recorded in a structured table, providing a comprehensive overview of study parameters. Descriptive statistical methods were applied to summarize trial characteristics, with categorical variables expressed as frequencies and percentages. For country-specific records, trials conducted in multiple countries were counted separately for each involved nation. Autoimmune diseases with low representation or trials covering multiple diseases were classified under “others.” Similarly, for cell type classification, uncommon, unidentified, or atypical stem cell types were grouped under “others.” Information on mechanisms of therapeutic action and therapeutic targets was extracted from the database fields “Primary Tested Drug: Mechanism of Action” and “Primary Tested Drug: Target”, respectively. A study-specific categorization system comprising eight mechanistic categories was developed to classify the mechanisms of therapeutic action (**Supplementary Table 1**). Trials involving multiple mechanisms were counted in each relevant category.

The therapeutic effects of stem cells derived from various sources on different autoimmune diseases were evaluated by categorizing clinical remission rates into three levels: low (≤50%), middle (>50% and ≤75%), and high (>75%), based on the tertile distribution of remission rates across all included trials. Only clinical studies with clearly reported remission rates were eligible for this categorization.

The information regarding drug combinations, which has been thoroughly disclosed, is categorized as follows: Conventional Treatment, HSC-based Multidrug Therapy, No Drug Combination, Other Multidrug Therapy, Biological Agents, Unclassified Drugs, Glucocorticoids, Cyclophosphamide, and Methotrexate. Conventional Treatment is defined as a range of standard therapies administered based on the specific autoimmune condition and patient requirements. HSC-based Multidrug Therapy refers to a therapeutic regimen involving the use of HSCs in combination with multiple drugs, which is uniformly administered to all patients. This therapy typically includes the administration of immunosuppressive agents such as cyclophosphamide, rituximab, and corticosteroids, alongside stem cell infusion. Other Multidrug Therapy refers to therapeutic regimens that combine stem cells from sources other than hematopoietic stem cells with various drugs, and these regimens are also uniformly applied to all patients.

Adverse events (AEs) were categorized by severity and their relationship to stem cell therapy into six groups. These included cases with no adverse events reported (No AE), events judged to be unrelated to the treatment (Unrelated AE), mild events where relatedness was not specified (Mild AE [Unspecified]), serious events with unspecified relatedness (Serious AE [Unspecified]), mild events considered related to the treatment (Mild AE [Related]), and serious events considered related to the treatment (Serious AE [Related]). All analyses were descriptive and conducted using GraphPad Prism (version 10.1.2).

## 3 Results

### 3.1 Trial characteristics

**Figure 1:**
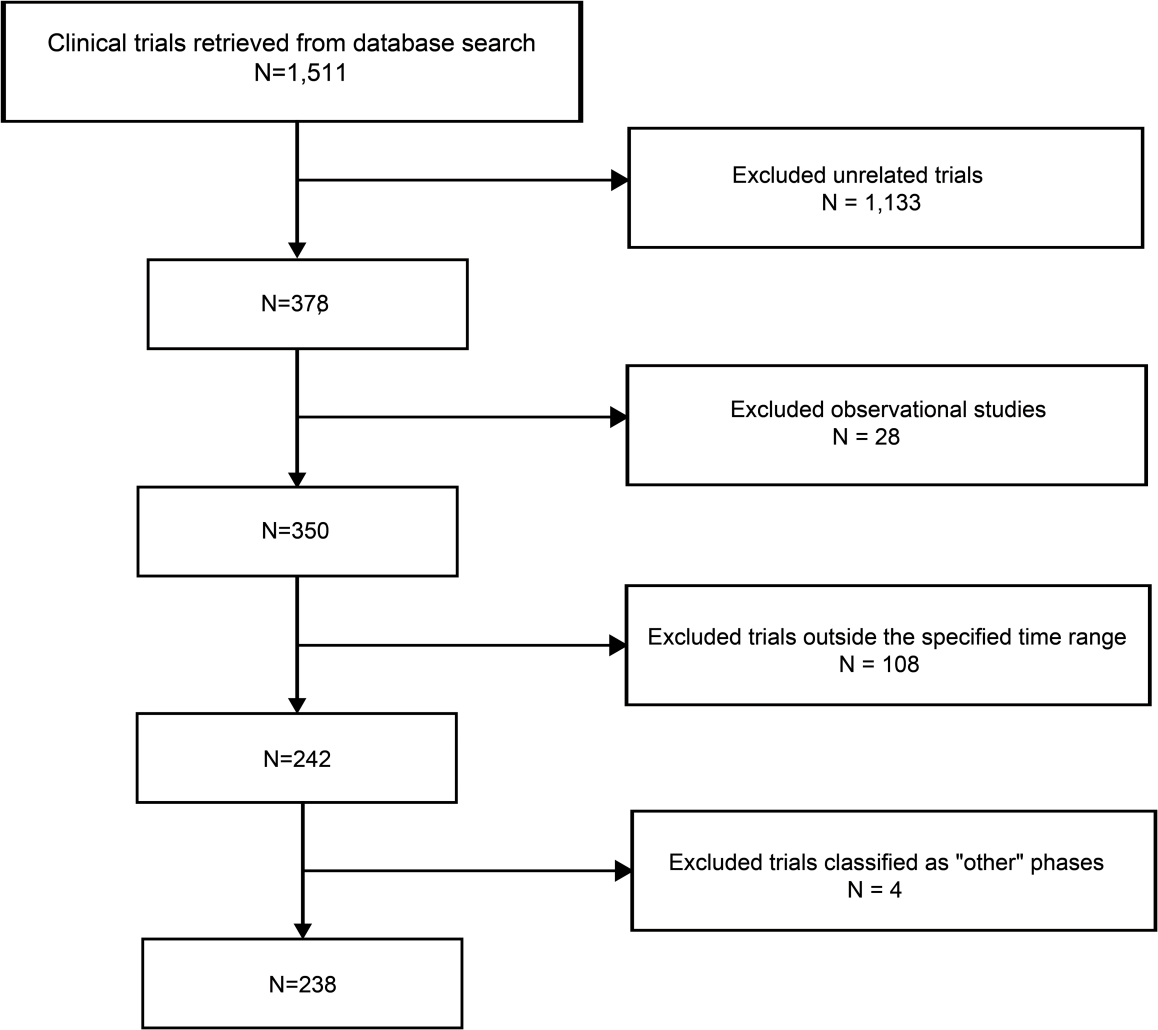
Flowchart of this study.

**Figure 2:**
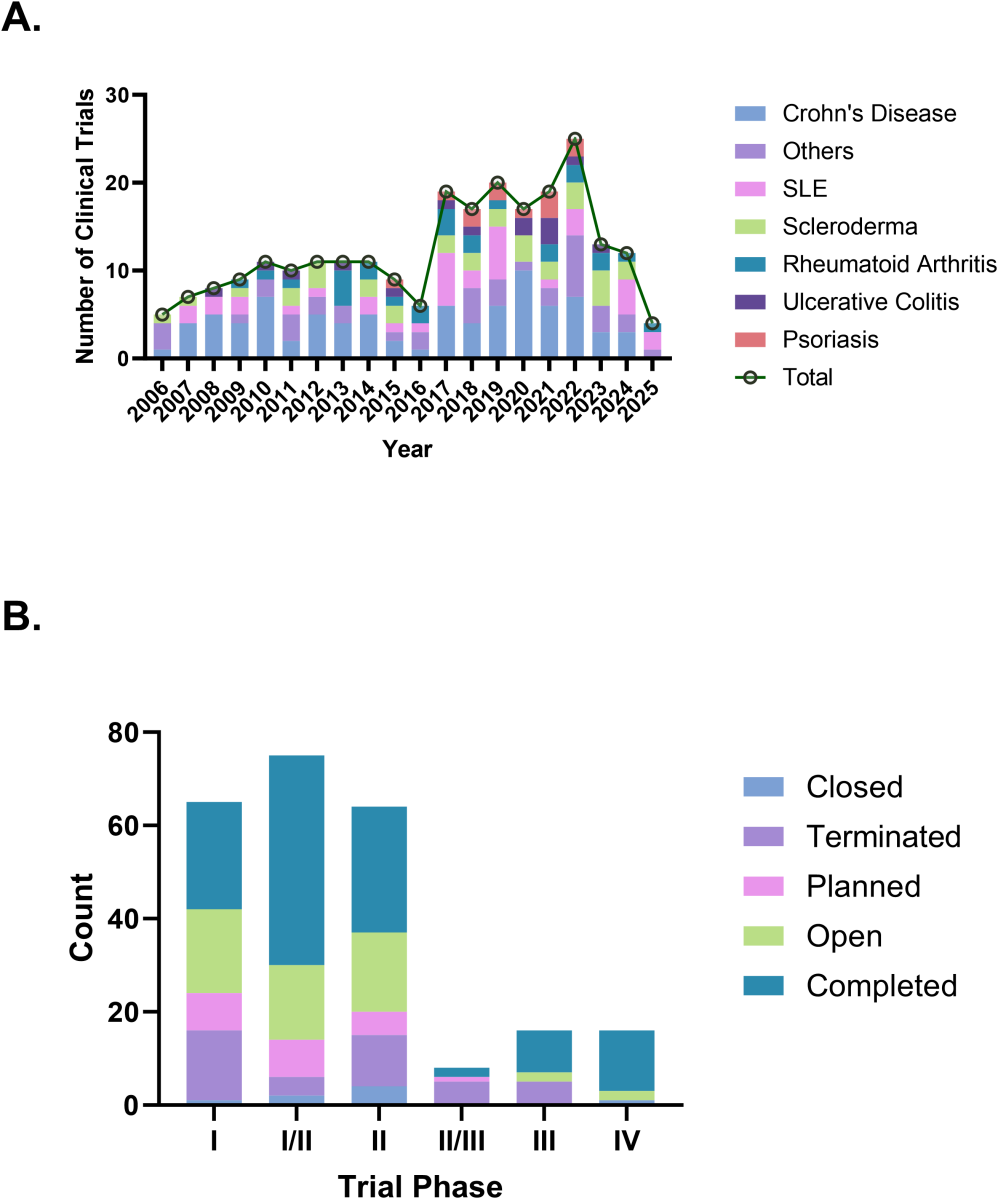
**(A)** Clinical trials trend over the years. **(B)** Clinical trials status by phase type.

As of January 2, 2025, a total of 1,511 stem cell therapy clinical trials in the “Autoimmune/Inflammation” field were registered globally. After screening, 1,133 trials that were unrelated to autoimmune diseases, 28 observational studies, 108 trials outside the specified time range, and 4 trials classified as falling under “other” phases were excluded, resulting in 238 trials included for analysis (**Figure 1**). We further cross-checked our results against ClinicalTrials.gov and the EU Clinical Trials Register, identifying six additional trials that were not captured in the Trialtrove database, bringing the final total to 244 trials. The number of clinical trials on autoimmune diseases has generally increased in recent years, with a notable peak in 2022, when the number of trials reached 25. In terms of disease distribution, CD had the highest number of trials (85 trials, 34.8%), followed by SLE (36 trials, 14.8%) and scleroderma (32 trials, 13.1%), while psoriasis had only 12 trials (**Figure 2A**). Across different clinical trial phases, most trials were in Phases I–II (204 trials, 83.6%), indicating that this field is still in the early exploratory stage of clinical development. A total of 40 trials were in Phases II–IV, with a completion rate of 60.0% (24 out of 40 trials) (**Figure 2B**), suggesting that while progress has been made, late-stage trials remain relatively limited.

### 3.2 Trial countries and funding sources

**Figure 3:**
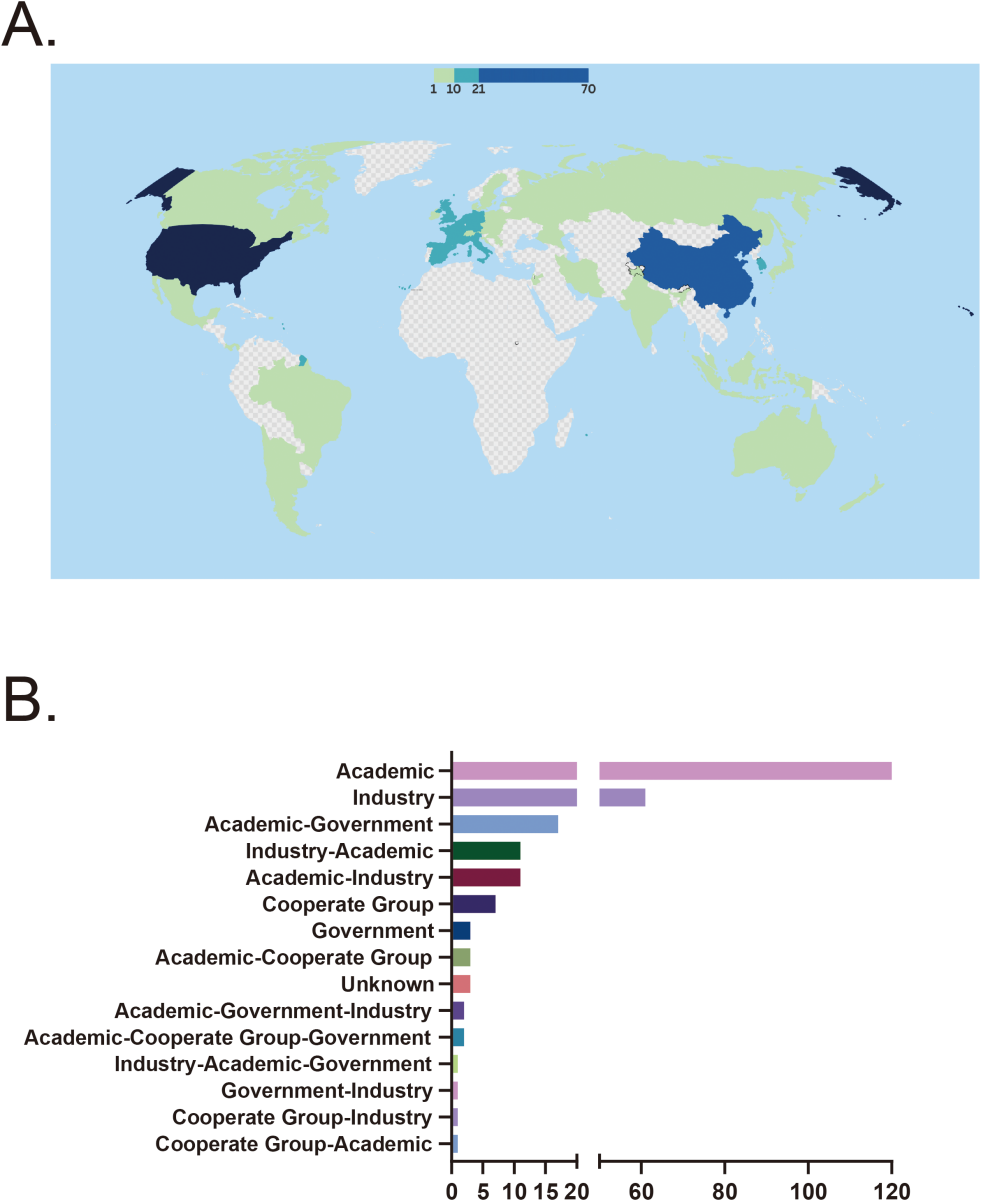
**(A)** Country distribution of clinical trials. **(B)** Distribution of funding sources. CD, Crohn’s disease; SLE, Systemic lupus erythematosus; RA, Rheumatoid arthritis; UC, Ulcerative colitis.

A total of 44 countries have conducted clinical trials on stem cell therapy for autoimmune diseases. The United States and China were the most active participants, with 70 trials (28.7%) in the U.S. and 62 trials (25.4%) in China. Countries such as Spain (19 trials, 7.8%), South Korea (15 trials, 6.1%), Belgium (13 trials, 5.3%), and France (14 trials, 5.7%) also demonstrated notable involvement. In contrast, 15 countries had only a single registered trial, indicating limited research activity in these regions (**Figure 3A**). Among all funding sources, academic institutions were the predominant contributors, supporting 120 trials (49.2%). Industry-sponsored trials also accounted for a significant proportion, totaling 62 trials (25.0%). However, government-funded trials were relatively rare, with only three recorded. In collaborative funding models, academic-industry partnerships funded 11 trials (4.5%), while academic-government collaborations supported 17 trials (7.0%). A small number of multisector collaborations, such as academic-government-industry partnerships and academic-cooperative group collaborations, funded a very limited number of trials, with no category exceeding three (**Figure 3B**).

### 3.3 Mechanisms of action and targets

**Figure 4:**
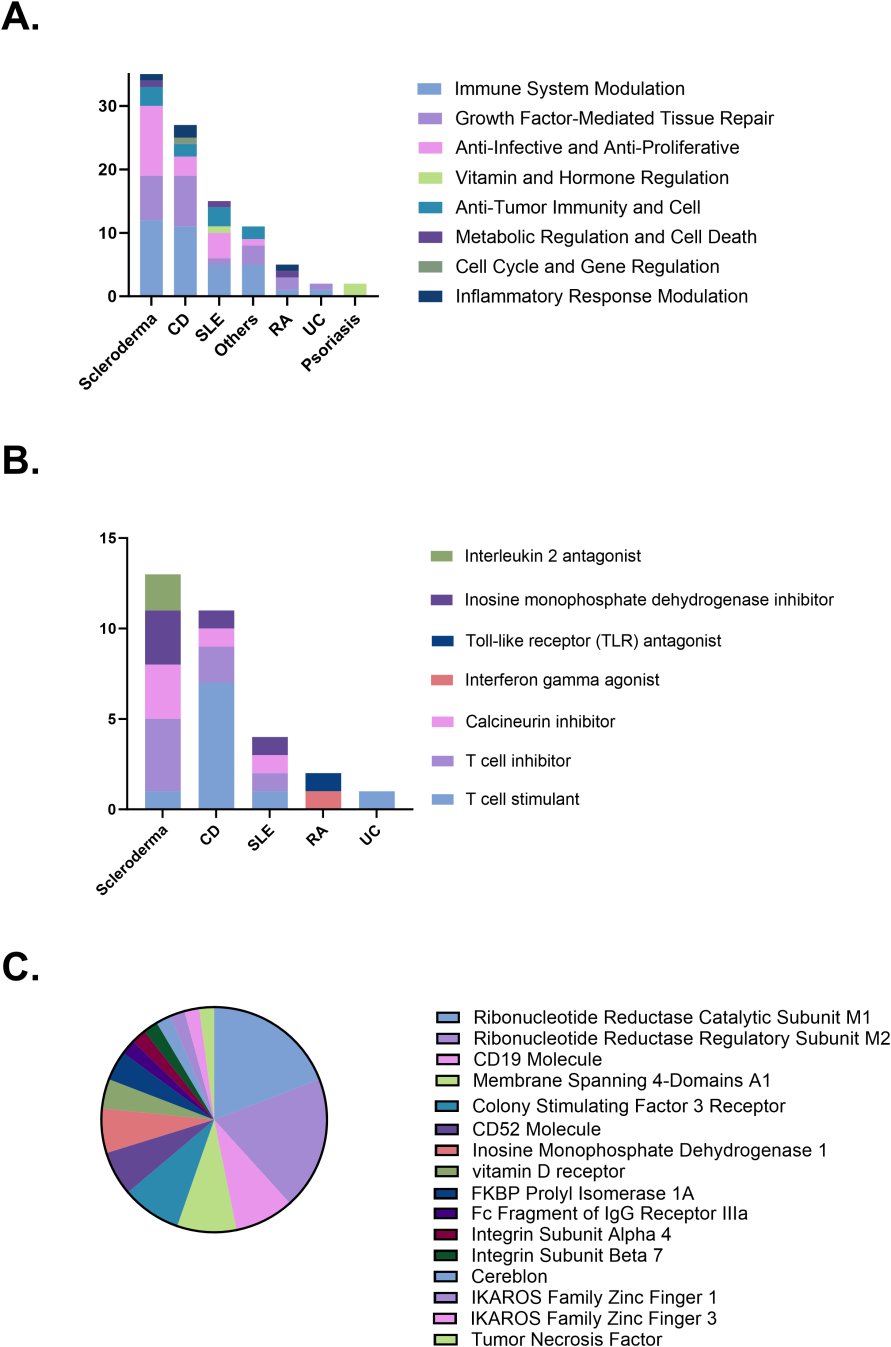
**(A)** Distribution of therapeutic mechanisms. **(B)** Immune modulation strategies by disease. **(C)** Therapeutic targets investigated in clinical trials. CD, Crohn’s disease; SLE, Systemic lupus erythematosus; RA, Rheumatoid arthritis; UC, Ulcerative colitis.

The mechanisms of therapeutic action in clinical trials primarily include immune system modulation (35 trials, 36.1%), growth factor-mediated tissue repair (22 trials, 22.7%), and anti-infective and anti-proliferative effects (19 trials, 19.6%). Different diseases prioritize distinct mechanisms. CD mainly involves immune system modulation, followed by growth factor-mediated tissue repair. Similarly, scleroderma primarily focuses on immune system modulation and anti-infective and anti-proliferative effects, a pattern also observed in SLE. In contrast, psoriasis clinical trials are predominantly associated with mechanisms involving vitamin and hormone regulation (**Figure 4A**). The specific strategies underlying immune system modulation also vary by disease type. Scleroderma is primarily associated with T cell inhibition, calcineurin pathway suppression, and inhibition of purine synthesis. Crohn’s disease is predominantly linked to T cell stimulation along with T cell inhibition. Systemic lupus erythematosus involves a diverse set of immune modulation strategies, with comparable frequencies across categories (**Figure 4B**). Regarding trial target studies, anti-tumor immunity and cell primarily involves molecules associated with immune responses, including CD19 (4 trials, 8.5%), membrane-spanning 4-domains A1 (4 trials, 8.5%), and CD52 (3 trials, 6.4%). The growth factor-mediated tissue repair mechanism primarily targets molecules related to cell proliferation and regeneration, such as colony-stimulating factor 3 receptor (4 trials, 8.5%). Anti-infective and anti-proliferative mechanisms predominantly target ribonucleotide reductase catalytic subunit M1 (RRM1; 9 trials, 19.1%) and ribonucleotide reductase regulatory subunit M2 (RRM2; 9 trials, 19.1%). Psoriasis clinical trials primarily investigate vitamin and hormone regulation mechanisms, with vitamin D receptor (2 trials) identified as the main target (**Figure 4C**).

### 3.4 Distribution characteristics of cell sources, donor types, and administration strategies

**Figure 5:**
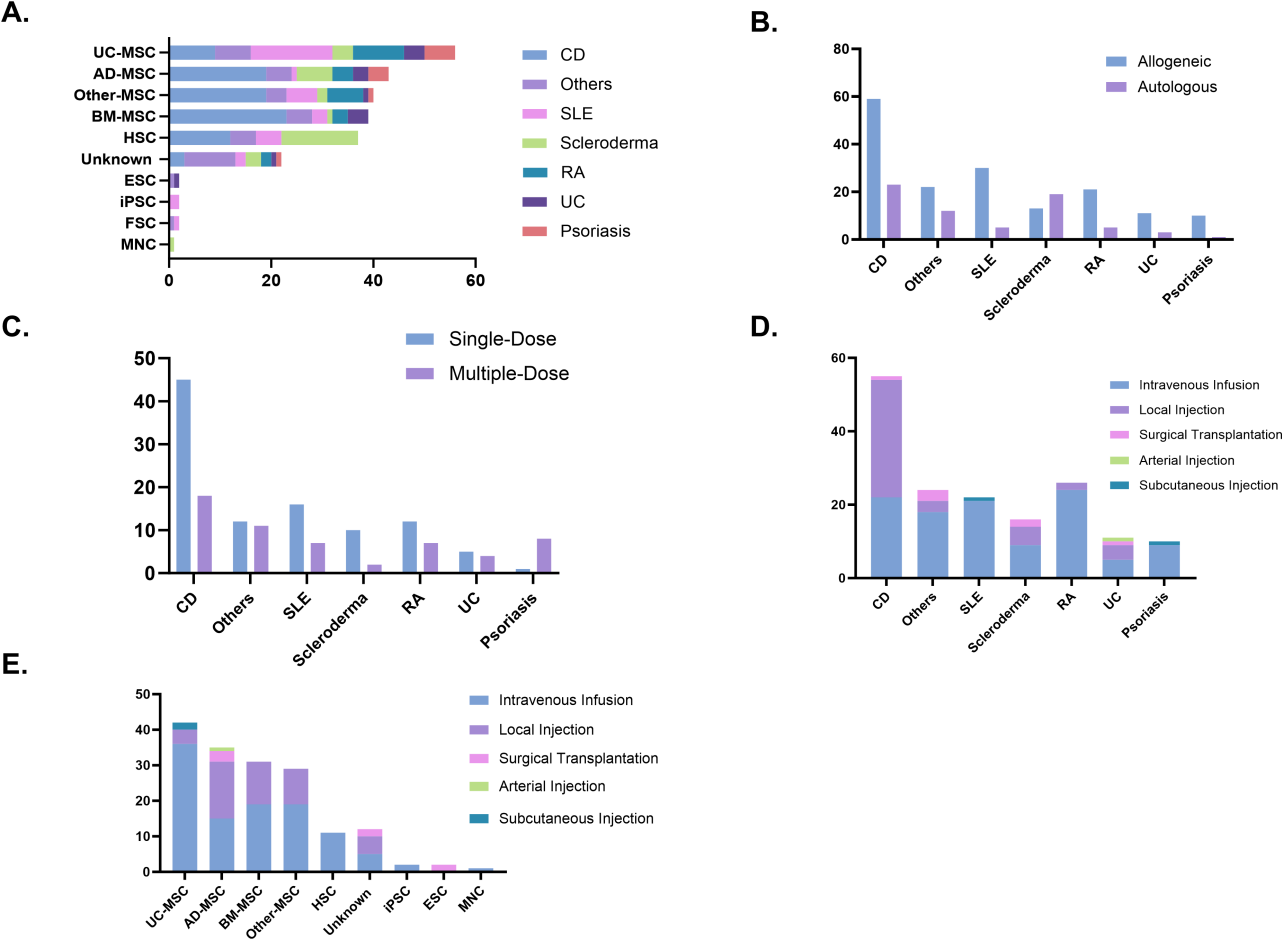
**(A)** Cell types used in clinical trials. **(B)** Donor types. **(C)** Dosing strategies. **(D)** Administration routes. **(E)** Administration routes by cell type. AD-MSC, adipose tissue-derived mesenchymal stem cells; BM-MSC, bone marrow-derived mesenchymal stem cells; CD, Crohn’s disease; ESC, embryonic stem cells; FSC, fetal stem cells; HSC, hematopoietic stem cells; iPSC, induced pluripotent stem cells; MNC, mononuclear cells; RA, rheumatoid arthritis; SLE, systemic lupus erythematosus; UC, ulcerative colitis; UC-MSC, umbilical cord-derived mesenchymal stem cells.

MSCs derived from bone marrow, adipose tissue, umbilical cord, and other sources accounted for the majority of cell types used in clinical trials (178 trials, 73.0%). Among these, CD was the most frequently studied condition, with Bone Marrow-derived Mesenchymal Stem Cells (BM-MSCs) being the most commonly applied. For SLE, stem cell therapy predominantly involved umbilical cord-derived mesenchymal stem cells (UC-MSCs). Similarly, HSCs were the primary choice for stem cell therapy in scleroderma. Additionally, other cell types, such as epithelial stem cells, mononuclear cells, iPSCs, and hair follicle stem cells, have also been explored in the treatment of autoimmune diseases, though their application remains less common (**Figure 5A**). In terms of stem cell donor sources, clinical trials on autoimmune diseases primarily used allogeneic stem cells (166 trials, 70.9%), while in scleroderma, autologous stem cells were more frequently utilized (19 trials, 59.3%) (**Figure 5B**). Treatment approaches varied across diseases. CD and scleroderma predominantly relied on single-dose stem cell therapy, whereas ulcerative colitis exhibited a more balanced distribution between single-dose and multiple-dose regimens. For psoriasis, treatment was mostly administered through repeated doses (**Figure 5C**). Regarding delivery methods, intravenous infusion was the predominant route of administration, particularly effective for systemic autoimmune diseases such as RA and lupus. In contrast, localized injection was preferred for gastrointestinal conditions such as CD. Surgical transplantation was rarely used and was primarily reserved for specific cases requiring targeted intervention (**Figure 5D**). In the cell-type stratification, Intravenous (IV) Infusion was overwhelmingly used in trials involving UC-MSC and HSC. A similar trend was observed for adipose-, bone marrow-, and other tissue-derived MSCs, where local injection also represented a notable proportion (**Figure 5E**).

### 3.5 Comparative evaluation of stem cell therapeutic efficacy, combination strategies, and safety profiles in autoimmune diseases

**Figure 6:**
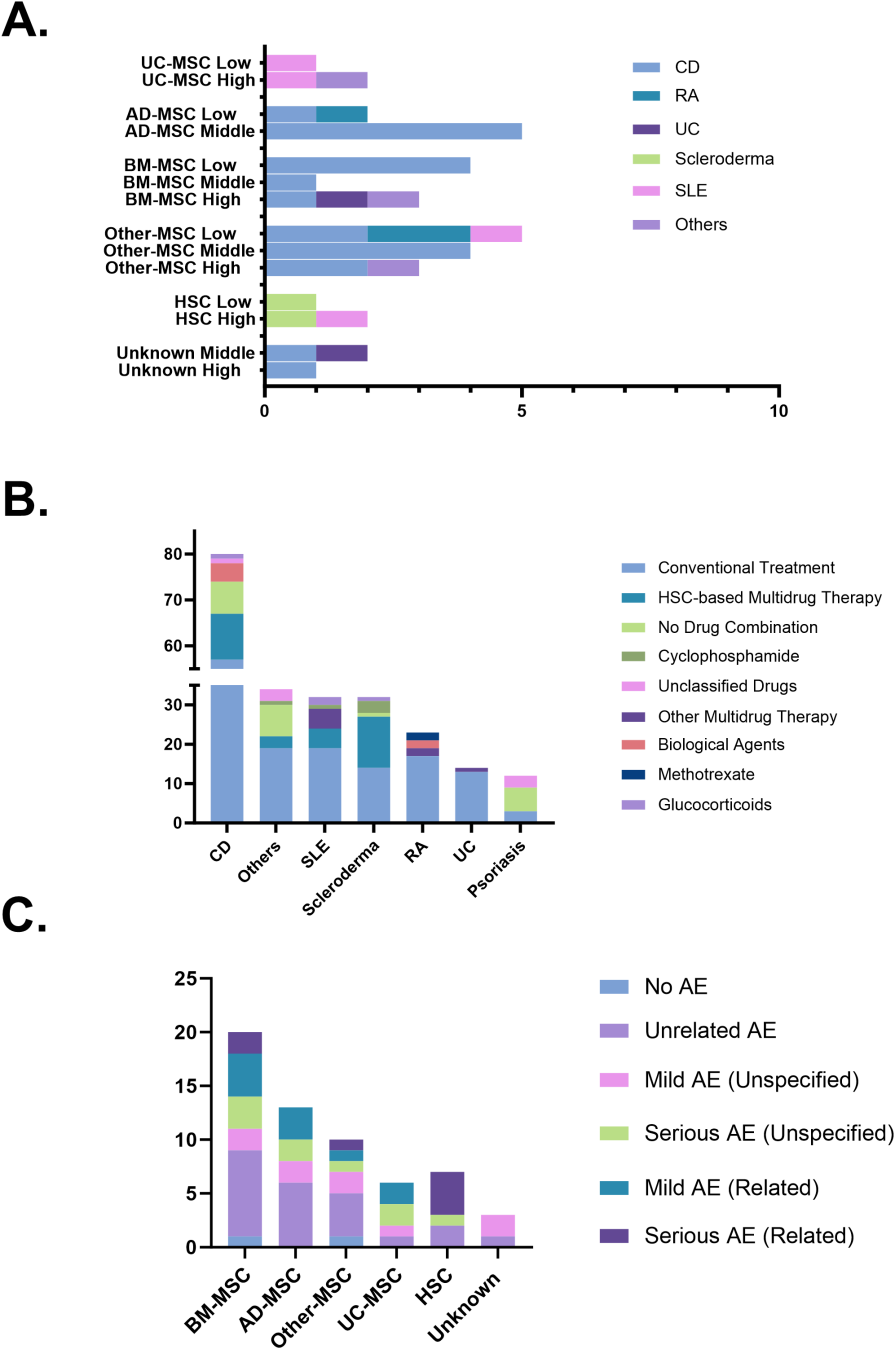
**(A)** Therapeutic efficacy across diseases and cell sources. **(B)** Combination strategies in stem cell therapy. **(C)** Safety profiles based on adverse event reports. AD-MSC, adipose tissue-derived mesenchymal stem cells; AE, adverse events; BM-MSC, bone marrow-derived mesenchymal stem cells; CD, Crohn’s disease; HSC, hematopoietic stem cells; MSC, mesenchymal stem cells; RA, rheumatoid arthritis; SLE, systemic lupus erythematosus; UC, ulcerative colitis; UC-MSC, umbilical cord-derived mesenchymal stem cells.

The therapeutic effects of stem cells derived from various sources on different autoimmune diseases were evaluated by categorizing clinical remission rates into low, middle, and high. Adipose-derived Mesenchymal Stem Cells (AD-MSC) and Other-MSC demonstrated consistent efficacy in treating CD, with the majority of clinical trials falling within the middle remission category. For RA, the efficacy of MSCs was relatively low. However, stem cell therapy achieved high efficacy across multiple other autoimmune diseases (**Figure 6A**). Regarding the drug combinations in stem cell therapy for autoimmune diseases, Conventional Treatment is the most commonly used approach (142trials, 62.6%), followed by Multidrug Therapy (39trials, 17.2%). Scleroderma shows a higher prevalence of HSC-based Multidrug Therapy, while Psoriasis and other autoimmune diseases exhibit a greater frequency of No Drug Combination. Biological Agents are used in CD and RA, while Glucocorticoids are used in CD, SLE, and Scleroderma (**Figure 6B**). A total of 59 adverse event (AE) records were extracted from completed clinical trials. The majority involved MSC products accounting for 49 out of 59 cases (83.1%). HSC products contributed 7 cases (11.9%). Unrelated AE remained the most frequently reported category (22 out of 59 cases, 37.3%). Among MSC-based trials, 17 mild and 11 serious adverse events were reported, accounting for 34.7% and 22.4% of MSC-related records, respectively. Of these, 10 mild (20.4%) and 3 serious (6.1%) adverse events were considered treatment-related. In contrast, HSC-based trials showed a notable concentration of treatment-related serious AEs, with 4 Serious AE (Related) reported, representing 57.1% (4/7) of all HSC-associated entries (**Figure 6C**).

## 4 Discussion

Over the past 20 years, CD and SLE have been the most actively studied autoimmune diseases in clinical trials of stem cell therapy, with a total of 85 trials for CD and 36 trials for SLE, respectively. The number of trials for CD reached its highest level in 2020, a trend that may be closely related to the growing clinical demand for effective intestinal mucosal repair strategies. In contrast, research on other autoimmune diseases, such as Sjögren’s syndrome and dermatomyositis, has been relatively limited, with only 4 and 7 trials conducted, respectively. This discrepancy highlights a notable research gap in the application of stem cell therapy for these conditions, suggesting the need for increased clinical focus and investment in underrepresented autoimmune diseases.

From the perspective of clinical trial phases, most studies are primarily concentrated in the early exploratory stage. Among Phase I trials, 23 out of 65 trials (35.4%) were successfully completed, while 15 trials (23.0%) were terminated. Potential reasons for trial discontinuation include technological limitations, such as immune rejection associated with allogeneic stem cells, as well as financial constraints and resource distribution issues. Notably, the high cost of autologous iPSC-derived therapies poses significant challenges in securing continuous funding (32). In Phase I/II trials, 45 out of 75 studies (60.0%) reached completion, suggesting that combined-phase designs facilitate dose selection optimization and allow for the preliminary validation of therapeutic mechanisms. For Phase III trials, 9 out of 16 studies (56.3%) were completed, whereas 5 trials (31.3%) were discontinued. This underscores the high barriers of late-stage trials, which require extensive patient cohorts and prolonged follow-up periods. Furthermore, these findings suggest that stem cell therapies face major challenges in large-scale validation, potentially due to the lack of standardization in cell preparation and concerns regarding treatment stability (33).

Standardization across multiple critical aspects of stem cell preparation is essential, as considerable variability arises from differences in cell sourcing, expansion, processing, and quality control procedures. Differences in cell sourcing, such as between bone marrow and adipose-derived cells, introduce variability due to distinct harvesting methods, cell yields, and biological characteristics. Variations in cell expansion protocols, particularly regarding passage number, can affect cell potency, as extended culturing often leads to diminished functional capacity associated with replicative senescence. Inconsistencies in processing methods, including cell isolation techniques, culture media composition, serum supplements, and oxygen conditions, further impact cell phenotype and therapeutic efficacy (34). Moreover, the absence of uniform quality control measures, including standardized assessments of viability, surface marker expression, functional potency, and safety parameters such as sterility and genomic stability, complicates comparisons across studies (35). Addressing these issues through the development of harmonized protocols is essential for improving the consistency of clinical outcomes and advancing the application of stem cell therapy in autoimmune diseases.

Stem cell therapy clinical trials in autoimmune diseases exhibit significant regional concentration. The United States leads in stem cell trials for autoimmune diseases (70 trials), reflecting robust federal support and clear regulatory pathways. The NIH has prioritized regenerative medicine through initiatives such as the Regenerative Medicine Innovation Project (RMIP) under the 21st Century Cures Act, which allocated USD 30 million for adult stem cell clinical research over 2017–2020 (36). Concurrently, the FDA regulates cell therapies as biological products, providing guidance on “Minimal Manipulation” and “Homologous Use” since 2017, while offering expedited review through the Regenerative Medicine Advanced Therapy designation (37). China’s stem cell trial landscape in autoimmune diseases (62 trials) is shaped by top-down strategies and evolving regulation. The 14th Five-Year Plan has emphasized regenerative medicine through substantial initiatives, including the launch of a ¥5 billion national innovation center. China adopts a dual-track regulatory system: hospital-based Investigator-Initiated Trials are overseen by the National Health Commission under the 2015 stem cell research measures, while industry-sponsored trials are regulated by the National Medical Products Administration under the Drug Administration Law (38). Beyond the US and China, other countries have developed unique stem cell trial frameworks. Spain has capitalized on its integrated national research networks and EU regulatory alignment to drive translational innovation in cell therapy. South Korea’s proactive regulatory reforms under the Ministry of Food and Drug Safety (MFDS), coupled with substantial government investment, have positioned it as a global frontrunner in advancing regenerative treatments (39). However, participation from other developing countries remains minimal. India, Brazil, and Argentina have each registered only one or two trials, while Africa has had no recorded involvement. This global disparity not only exacerbates healthcare inequalities but may also skew therapy development toward the disease profiles of high-income countries (40). Consequently, the research needs of immune diseases that are more prevalent in tropical regions, such as certain regional subtypes of RA that remain underexplored in African populations, may be overlooked in global therapy development (41). In terms of funding sources, academic institutions play a dominant role in stem cell therapy research, independently conducting 120 trials (49.2%). However, cross-sector collaborations remain scarce. Academic-industry partnerships account for only 11 trials, while government-industry collaborations are limited to just one trial. This lack of interdisciplinary cooperation may hinder the efficient translation of technology. Innovative academic research findings often struggle to transition into clinical applications due to insufficient industry engagement, while industry-driven demands, such as the need for scalable manufacturing processes, are not adequately integrated into fundamental research.

CD19 and CD52, as critical targets for Immuno-oncology therapy, have demonstrated unique value in stem cell therapies for autoimmune diseases (42, 43). CD19, a surface marker of B cells, plays a key role in immune regulation by targeting and clearing abnormally activated B cells or inhibiting their function, thereby demonstrating significant efficacy in diseases such as SLE. For example, FT819 (NCT06308978), an iPSC-based allogeneic CAR-T cell therapy, is designed to target CD19 for the depletion of pathogenic B cells in SLE patients and is currently in a Phase I clinical trial. CD52’s broad-spectrum immune-clearing properties make it a promising target for multiple diseases. For example, in the NCT00692939 trial, the treatment approach combines Alemtuzumab, which depletes CD52-positive immune cells such as T cells and B cells, with autologous CD34+ stem cell transplantation to create an immune-silent environment and promote immune system reconstruction. This strategy aims to improve clinical symptoms in severe CD patients, particularly those with refractory intestinal fistulas. In addition to these frequently targeted molecules, other immune-related targets have also been investigated in stem cell-based clinical trials. For example, CD20 is indirectly represented in our dataset through the gene MS4A1 (4 trials, 8.5%), reinforcing the central role of B cell depletion (44). Within the growth factor-mediated tissue repair mechanism, a noteworthy target is the colony-stimulating factor 3 receptor, which facilitates hematopoietic stem cell mobilization (45). Anti-infective and anti-proliferative effects are commonly observed in conditioning regimens prior to hematopoietic stem cell transplantation. Notably, RRM1 and RRM2, each recorded in 9 trials (19.1%), are key targets associated with DNA synthesis and cell proliferation. Their involvement reflects the role of pre-transplant cytotoxic strategies in suppressing overactive immune components. Psoriasis trials in our dataset were predominantly linked to mechanisms involving vitamins or hormones, with the vitamin D receptor emerging as a key target in a couple of studies. This focus aligns with the known immunoregulatory role of vitamin D in skin autoimmunity (46). It is worth noting that several well-established therapeutic targets, widely recognized in the field of autoimmune research, were not identified in our current dataset. These include B cell-associated molecules such as CD22, BAFF, and APRIL, which are critical for B cell survival and have been targeted by monoclonal antibodies like belimumab and atacicept (44). Similarly, key T cell activation and co-stimulatory markers such as CD3, CD25, CD28, and CD40/CD40L, as well as immune checkpoint molecules including PD-1 and PD-L1, were absent from our included trials. Likewise, pathways involved in immune cell migration, including integrins like VLA-4 and sphingosine-1-phosphate receptors, were not specified, despite their relevance in autoimmune pathogenesis. In terms of regenerative mechanisms, growth factors such as VEGF, HGF, IGF-1, FGF2, and SCF/c-Kit were not identified as direct targets, although they are known to be secreted by mesenchymal stem cells and contribute to tissue repair (47). These observations suggest that future trials may benefit from integrating a broader array of immune, inflammatory, regenerative, and metabolic targets to fully exploit the multifaceted therapeutic potential of stem cell-based interventions.

Routes of administration for stem cell therapies vary depending on disease context and cell type, reflecting differences in therapeutic objectives and cellular properties. MSCs have been administered via various routes, including intravenous (IV), intraperitoneal, subcutaneous, and direct local injection, with IV infusion reported as the most common delivery method (48). The higher proportion of IV infusion observed for UC-MSCs, compared with AD- or BM-derived MSCs, likely reflects their stronger immunomodulatory capacity and comparatively lower immunogenicity (49). In contrast, adult tissue-derived MSCs are frequently employed for localized tissue repair requiring targeted delivery, as they originate from mature tissues and possess greater regenerative and structural repair potential (50). HSCT, in clinical trials for autoimmune diseases, is uniformly administered via intravenous infusion. Although the intrabone marrow route has been investigated in preclinical and early clinical studies, it has not shown clear advantages over IV infusion in terms of long-term engraftment or hematopoietic reconstitution (51). Intravenous infusion remains the predominant method for therapies derived from iPSCs, particularly CAR-engineered immune cells. Conversely, for epithelial stem cells, such as corneal epithelial stem cells and intestinal epithelial stem cells, direct surgical transplantation is preferred. By improving the delivery method, particularly through Intravenous Infusion, stem cell therapy for autoimmune diseases has gained new momentum. A recent study introduced a method of integrating dexamethasone liposomes into mesenchymal stem cells (Dexlip-MSCs), which not only extended the circulation time of dexamethasone liposomes *in vivo* but also effectively activated the glucocorticoid receptor signaling pathway. This activation occurred through the inhibition of CD4^+^ T cell proliferation and the release of pro-inflammatory mediators, while simultaneously upregulating the expression of anti-inflammatory factors. As a result, Dexlip-MSCs demonstrated superior efficacy in an SLE animal model when compared to either Dexlip or MSC treatment alone. Additionally, employing injectable hydrogel technology for sustained-release applications of exosomes has shown potential to extend the therapeutic effect and reduce the number of infusions (52, 53).

Across the aggregated dataset, stem-cell products of disparate origins yielded broadly comparable remission profiles, and no source-dependent superiority could be discerned within the descriptive scope of the present analysis. Nevertheless, source-specific therapeutic profiles become more nuanced when considered within the context of each autoimmune indication. HSCT confers the most robust disease-modifying benefit in systemic sclerosis, with sustained skin and lung improvement and superior event-free survival; meanwhile, MSC therapy has demonstrated significant reductions in skin thickness, digital ulcers, and hand pain, along with improvements in lung function over 12 months (54). In SLE, HSCT performed in experienced centres yields 68–86% progression-free survival at ten years, whereas allogeneic MSC infusion achieves complete or partial remission in roughly half of refractory cases, and amniotic epithelial cells have reversed nephritis in murine models (55). RA saw early HSCT induce six-month remission in about 60% of end-stage patients before frequent relapse curtailed its use, while I/II MSC studies (NCT01851070) demonstrated promising short-term efficacy in biologic-refractory RA, with ACR70 responses achieved in 27% and ACR50 in 31% of patients receiving the higher dose at 12 weeks, compared to 0% and 19% in the placebo group, respectively. In Crohn’s disease, HSCT offers limited durable benefit and appreciable risk, while local injection of allogeneic adipose-derived MSCs achieved a 76% complete healing rate in perianal fistulas (TrialTroveID-429449), and pre-clinical work combining induced-PSC MSCs with intestinal epithelial organoids restores mucosal integrity (56). Ulcerative colitis rarely warrants HSCT because surgical colectomy is curative, yet two monthly infusions of umbilical-cord MSCs induce 41% clinical remission at eight weeks with one-third mucosal healing (57).

In clinical trials targeting autoimmune diseases, MSCs derived from adult or perinatal tissues constitute the predominant cell type. Their widespread application is largely attributed to their potent immunomodulatory properties, favorable safety profile with low tumorigenicity, and capacity for allogeneic use due to immune-evasive characteristics (58, 59). HSCs are primarily applied in severe, refractory autoimmune diseases where immune ablation followed by reconstitution is necessary to achieve durable remission, though their use is limited by higher treatment-related risks (60). Pluripotent stem cells such as embryonic stem cells (ESCs) and iPSCs are rarely used in this context due to concerns regarding tumorigenicity, ethical considerations, and the need for directed differentiation to obtain functionally relevant cell types (58, 61). Mononuclear cells and other less commonly used cell types are still under exploration and currently represent only a small fraction of ongoing clinical investigations. MSCs from different sources exhibit distinct clinical applicability, with BM-MSCs being particularly prevalent in clinical trials for CD treatment. A recent open-label trial assessed the safety and efficacy of BM-MSCs in patients with penetrating perianal CD. The study included 16 patients who underwent localized BM-MSC injections. The results indicated that after receiving MSC treatment, 9 out of 16 patients achieved complete fistula closure by week 12, with no serious adverse events reported. Although AD-MSCs are more commonly used in this indication, this preliminary evidence supports BM-MSCs as a promising alternative for CD treatment (62). On the other hand, previous high-dose immunoablation HSCT protocols in refractory CD have demonstrated limited efficacy and were associated with serious adverse events. The ASTIClite trial introduced a new strategy featuring low-dose mobilization and reduced-intensity preconditioning. At week 48, 43% of patients achieved the endpoint of complete ulcer healing, demonstrating significant progress in improving lesion activity through HSCT (63). UC-MSCs, recognized for their multi-differentiation potential and significant immunomodulatory effects, have emerged as a key option in clinical trials for SLE. A recent study developed a single-cell atlas of human UC-MSCs through large-scale single-cell transcriptome analysis, identifying three cell populations (C1, C2, and C3), which correspond to pre-activation, transitional, and stemness states. This finding not only elucidates the inherent heterogeneity of UC-MSCs but also provides a new perspective for understanding their mechanism of action in SLE treatment (64). Future efforts should focus on employing multi-omics techniques to further analyze the functional differences of UC-MSC subpopulations and refine biomarker-based cell sorting methods. These advancements may drive the clinical translation of UC-MSC-based precision therapy, ultimately offering safer and more effective treatment options for SLE patients. HSCT has been established as the primary option in stem cell therapy for scleroderma, particularly for managing the severe complication of gastric antral vascular ectasia (GAVE). A recent retrospective study showed that among scleroderma patients who underwent HSCT, those with GAVE achieved complete remission both endoscopically and histologically, with satisfactory safety profiles. Compared to therapies such as cyclophosphamide, HSCT not only improved scleroderma-related vascular lesions but may also provide long-lasting effects (65).

In this study, we systematically collected and screened stem cell therapy studies focused on autoimmune diseases at various stages from global clinical trial databases, ultimately including a total of 244 trials for comprehensive analysis. The included trials span various types of stem cells and encompass multiple immune-related diseases, including CD, SLE, and scleroderma. The significance of this study lies in its multifaceted and systematic review of the current state and trends of stem cell therapy in autoimmune diseases, highlighting the differences and similarities in stem cell sources, delivery methods, and trial designs. These findings provide evidence-based support for future clinical decisions and the standardization of stem cell preparation. However, certain limitations should be acknowledged. Inherent limitations such as occasional data omissions and uneven regional coverage may still result in gaps in the available research data. In addition, heterogeneity across trials, including differences in patient selection criteria, disease severity, outcome definitions, follow-up duration, and reporting standards, may have introduced inconsistencies during data synthesis. Secondly, the classification method for diseases and cell types could lead to data oversimplification. For example, rare autoimmune diseases with varying clinical presentations were grouped together as “others” in this study, which may have masked disease-specific variations, making it difficult to explore the potential value of small-sample diseases in stem cell therapy.

The future prospects for stem cell therapy in autoimmune diseases are promising, with the key factors lying in technological innovation and the development of engineered stem cells. With the continuous advancement of gene editing technologies such as CRISPR-Cas9, we can enhance functional research and dynamic monitoring of stem cells by precisely integrating multimodal reporter genes (66). In addition, constructing a perinatal stem cell bank is expected to become an important strategy to address the limitations of autologous stem cell function. By drawing on Japan’s successful experience with the “iPS Cell Bank,” this technology may be further promoted through international cooperation (67). In precision medicine, the application of standardization and multi-omics technologies will significantly enhance data comparability and improve the ability to predict treatment efficacy. In addition to applying single-cell transcriptomics to quantitatively assess specific cell subpopulations and reveal differences between treatment protocols, future advancements may utilize liquid biopsy technologies to monitor dynamic biomarkers such as exosomal miRNA in real-time, enabling more precise and individualized tracking of treatment responses in autoimmune disease patients (68). Moreover, by integrating a broad range of indicators such as gut microbiota diversity and HLA typing, patients can be precisely stratified. This approach is expected to provide customized stem cell intervention strategies tailored to the needs of patients with CD (69, 70). At the same time, relying on large-scale, multi-center real-world data, researchers can comprehensively evaluate the efficacy and safety of stem cell therapy in clinical practice. This data-driven approach can also reveal key prognostic factors influencing treatment response, thereby driving the intelligent design and precise optimization of stem cell therapy protocols (71).

Overall, breakthroughs in combination therapies, technological innovations, international collaboration, and precision medicine will collectively accelerate the clinical translation and widespread adoption of stem cell therapies for autoimmune diseases, ultimately providing patients with safer, more effective, and cost-efficient treatment options.

## 5 Conclusion

Stem cell therapies show great promise in autoimmune diseases. Global clinical trial data indicate that CD, systemic lupus erythematosus, and scleroderma are the most active areas of research, but most trials are still in early stages and are highly concentrated geographically. Mechanistically, immune regulation, tissue repair, and anti-cell proliferation are the main intervention targets. However, stem cell therapies still face many challenges in clinical translation for autoimmune diseases: high costs of personalized treatments, lack of long-term safety data, and insufficient standardization of cell preparation. The future must focus on technological innovation, international collaboration, and precision medicine to overcome these challenges.

## Data Availability

The original contributions presented in the study are included in the article/**Supplementary Material**. Further inquiries can be directed to the corresponding authors.
