# Supplementary material for: Global clinical trials on stem cell therapy for autoimmune diseases: trends and future directions
Source: Front Immunol. 2025 Jul 24;16:1616231. doi: 10.3389/fimmu.2025.1616231 (PMC12328461; doi:10.3389/fimmu.2025.1616231)
Supplement: Supplementary file 1 [file Table1.docx]

# Supplemental Table 1

# Therapeutic Mechanism Categorization

| Category | Mechanism Term |
| --- | --- |
| Immune System Modulation | Calcineurin inhibitor |
| Immune System Modulation | Immunostimulant |
| Immune System Modulation | Immunosuppressant |
| Immune System Modulation | Inosine monophosphate dehydrogenase inhibitor |
| Immune System Modulation | Interferon gamma agonist |
| Immune System Modulation | Interleukin 2 antagonist |
| Immune System Modulation | Interleukin 2 receptor agonist |
| Immune System Modulation | T cell inhibitor |
| Immune System Modulation | T cell stimulant |
| Growth Factor-Mediated Tissue Repair | Angiogenesis stimulant |
| Growth Factor-Mediated Tissue Repair | Collagen stimulant |
| Growth Factor-Mediated Tissue Repair | Fibrinogen stimulant |
| Growth Factor-Mediated Tissue Repair | Granulocyte colony stimulating factor agonist |
| Growth Factor-Mediated Tissue Repair | Stem cell growth factor agonist |
| Anti-Infective and Anti-Proliferative | Cell wall synthesis inhibitor |
| Anti-Infective and Anti-Proliferative | DNA inhibitor |
| Anti-Infective and Anti-Proliferative | DNA repair enzyme inhibitor |
| Anti-Infective and Anti-Proliferative | DNA synthesis inhibitor |
| Anti-Infective and Anti-Proliferative | Microtubule inhibitor |
| Anti-Infective and Anti-Proliferative | Ribonucleoside diphosphate reductase inhibitor |
| Vitamin and Hormone Regulation | Corticosteroid agonist |
| Vitamin and Hormone Regulation | Glucocorticoid agonist |
| Vitamin and Hormone Regulation | Vitamin D agonist |
| Anti-Tumor Immunity and Cell | Angiogenesis inhibitor |
| Anti-Tumor Immunity and Cell | CD20 antagonist |
| Anti-Tumor Immunity and Cell | CD52 antagonist |
| Anti-Tumor Immunity and Cell | CD71 antagonist |
| Anti-Tumor Immunity and Cell | Immuno-oncology therapy |
| Anti-Tumor Immunity and Cell | Natural killer cell stimulant |
| Metabolic Regulation and Cell Death | Apoptosis stimulant |
| Metabolic Regulation and Cell Death | Inosine monophosphate dehydrogenase inhibitor |
| Cell Cycle and Gene Regulation | Cereblon E3 ubiquitin ligase stimulant |
| Cell Cycle and Gene Regulation | IKAROS family zinc finger 1 inhibitor |
| Cell Cycle and Gene Regulation | IKAROS family zinc finger 3 inhibitor |
| Cell Cycle and Gene Regulation | Protein degrader |
| Inflammatory Response Modulation | Alpha4β7 integrin antagonist |
| Inflammatory Response Modulation | Arachidonic acid inhibitor |
| Inflammatory Response Modulation | Toll-like receptor (TLR) antagonist |
| Inflammatory Response Modulation | Tumour necrosis factor alpha antagonist |
